# Dynamic Changes of the Neutrophil-to-Lymphocyte Ratio, Systemic Inflammation Index, and Derived Neutrophil-to-Lymphocyte Ratio Independently Predict Invasive Mechanical Ventilation Need and Death in Critically Ill COVID-19 Patients

**DOI:** 10.3390/biomedicines9111656

**Published:** 2021-11-10

**Authors:** Emanuel Moisa, Dan Corneci, Silvius Negoita, Cristina Raluca Filimon, Andreea Serbu, Mihai Ionut Negutu, Ioana Marina Grintescu

**Affiliations:** 1 Department of Anaesthesia and Intensive Care Medicine, Faculty of Medicine, ‘Carol Davila’ University of Medicine and Pharmacy, 020021 Bucharest, Romania; dan.corneci@umfcd.ro (D.C.); silviusneg@gmail.com (S.N.); ioana.grintescu@rospen.ro (I.M.G.); 2 Clinic of Anaesthesia and Intensive Care Medicine, Elias Emergency University Hospital, 011461 Bucharest, Romania; mnegutu@gmail.com; 3 Clinic of Anaesthesia and Intensive Care Medicine, Dr. Carol Davila Central Military Emergency University Hospital, 010825 Bucharest, Romania; filimon_raluca@yahoo.ro (C.R.F.); andreea.serbu91@gmail.com (A.S.); 4 Clinic of Anaesthesia and Intensive Care Medicine, Clinical Emergency Hospital of Bucharest, 014461 Bucharest, Romania

**Keywords:** COVID-19, NLR, systemic inflammation index, dNLR, PLR, MLR, sepsis, mechanical ventilation, ICU

## Abstract

Background: Hematological indices can predict disease severity, progression, and death in patients with coronavirus disease-19 (COVID-19). Objectives: To study the predictive value of the dynamic changes (first 48 h after ICU admission) of the following ratios: neutrophil-to-lymphocyte (NLR), platelet-to-lymphocyte (PLR), monocyte-to-lymphocyte (MLR), systemic inflammation index (SII), and derived neutrophil-to-lymphocyte (dNLR) for invasive mechanical ventilation (IMV) need and death in critically ill COVID-19 patients. Methods: Observational, retrospective, and multicentric analysis on 272 patients with severe or critical COVID-19 from two tertiary centers. Hematological indices were adjusted for confounders through multivariate analysis using Cox regression. Results: Patients comprised 186 males and 86 females with no difference across groups (*p* > 0.05). ΔNLR > 2 had the best independent predictive value for IMV need (HR = 5.05 (95% CI, 3.06–8.33, *p* < 0.0001)), followed by ΔSII > 340 (HR = 3.56, 95% CI 2.21–5.74, *p* < 0.0001) and ΔdNLR > 1 (HR = 2.61, 95% CI 1.7–4.01, *p* < 0.0001). Death was also best predicted by an NLR > 11 (HR = 2.25, 95% CI: 1.31–3.86, *p* = 0.003) followed by dNLR > 6.93 (HR = 1.89, 95% CI: 1.2–2.98, *p* = 0.005) and SII > 3700 (HR = 1.68, 95% CI: 1.13–2.49, *p* = 0.01). Conclusions: Dynamic changes of NLR, SII, and dNLR independently predict IMV need and death in critically ill COVID-19 patients.

## 1. Introduction

The severe acute respiratory syndrome coronavirus 2 (SARS-CoV-2) pandemic is an ongoing public health issue affecting millions of people throughout the world. Despite recent advances (e.g., monoclonal antibodies therapy, specific vaccines) that appear to be decreasing hospitalization and mortality rates [1,2], severe or critical cases that require ICU (intensive care unit) admission are still an important burden.

Simple tools as markers of disease severity and progression are still under investigation. These could be used in patients’ triage, especially those requiring ICU admission, respiratory support, or in whom death rates are high irrespective of provided medical care. In severe coronavirus disease-2019 (COVID-19), the lungs are mainly involved and the patients present with respiratory failure [3], which can progress to critical illness defined as acute respiratory distress syndrome (ARDS), sepsis, or septic shock with subsequent respiratory failure [4]. In fact, critical patients with COVID-19 have a type of viral sepsis [4,5]. Of a cohort of 4404 patients admitted to a hospital in New York, 9 out of 100 patients admitted required ICU admission upon arrival and 12 out of 100 required ICU admission in the next 2–3 days [6]. In a meta-analysis conducted by Abate et al., ICU admission for hospitalized patients was 32–40% with mortality rates as high as 86% [7].

Hematological parameters are measured routinely in critical patients using complete blood count analysis. Lymphopenia, neutrophilia, and leukocytosis are frequently reported in COVID-19 patients, being correlated with disease severity and prognosis [8,9,10,11]. Disturbances in eosinophil and platelet count are also observed in these patients [12,13,14]. Furthermore, derived hematological indices like the neutrophil-to-lymphocyte ratio (NLR), platelet-to-lymphocyte ratio (PLR), monocyte-to-lymphocyte ratio (MLR), systemic inflammation index (SII), and derived neutrophil-to-lymphocyte ratio (dNLR) were correlated with disease severity or progression [10,12,13,14] and mortality [12,13,15]. Since COVID-19’s emergence, different cut-off values have been reported for disease severity and death, but a standard cut-off value for each hematological index has not yet been established.

Therefore, our objectives were to study the predictive value of different hematological parameters measured on routine complete blood count and their derived hematological indices at ICU admission (0 h), 48 h, and their dynamic change (delta values) in critically ill patients with COVID-19. Our primary end-points were the predictive value of these indices for the need of invasive mechanical ventilation during length of stay and death.

## 2. Materials and Methods

### 2.1. Study Population

We conducted an observational, retrospective, and multicentric analysis on a cohort of 272 patients with severe or critical COVID-19 pneumonia admitted to four intensive care units from two tertiary centers: Elias University Emergency Hospital and Carol Davila Central Military University Emergency Hospital in Bucharest, Romania during a period of 11 months (from 1 April 2020 to 28 February 2021). Each hospital had an ICU department but also a modular intensive care unit in a different location. Local ethics committees from both hospitals approved the study. The inclusion criteria were age between 18 and 85, acute respiratory failure requiring non-invasive or invasive mechanical ventilation or high-flow oxygen therapy, COVID-19 pneumonia confirmed through real time-polymerase chain reaction (RT-PCR) and CT-scan or chest X-ray, and ICU length of stay (ICU-LOS) ≥ 72 h. The exclusion criteria were age over 85, advanced oncological disease or ongoing radio-, chemo- or immunotherapy, patients with confirmed or suspected bacterial associated infection (positive bacterial cultures, procalcitonin > 2 ng/mL, radio-imagistic findings suggestive for bacterial superinfection), end-stage organ diseases (liver, cardiac, kidney), ICU-LOS < 72 h or death in the first 72 h, patients transferred from other hospitals and intubated for >48 h and moderate-severe autoimmune diseases.

### 2.2. Clinical, Hematological, and Biochemical Examinations

We collected demographic data (age, gender, body mass index), data about associated diseases (cardiac disease, respiratory disease, diabetes mellitus, chronic kidney disease, liver disease), the Charlson Comorbidity Index, SOFA (Sequential Organ Failure Assessment) score, and hematological and biochemical parameters at ICU admission and at 48 h as follows: absolute leukocyte count, absolute neutrophil count, absolute lymphocyte count, absolute monocyte count, absolute platelet count, immature granulocytes count, D-dimers, C-reactive protein (CRP), ferritin, and P/F ratio. Also, data about respiratory status at ICU admission (invasive or non-invasive mechanical ventilation, high-flow oxygen therapy) and progression to endotracheal intubation and invasive mechanical ventilation (IMV), the day of endotracheal intubation and IMV initiation, length of stay (LOS) and survival, patients who received anti-inflammatory therapy with corticosteroids, and immunotherapy with tocilizumab (RoActemra^®^, Roche GmbH, Mannheim, Germany) or anakinra (Kineret^®^, Swedish Orphan Biovitrum AB, Stockholm, Sweden) and/or antiviral treatment with remdesivir (Veklury^®^, Gilead, Foster City, CA, USA) in the first 36 h were collected. 

### 2.3. Data Analysis

Data analysis was conducted depending on the main two events studied: need for IMV and death. For the first event mentioned, IMV, patients were divided in 3 groups: group 1—patients on IMV upon ICU admission, group 2—patients that required IMV during ICU-LOS, and group 3—patients non-IMV during ICU-LOS. For the death event, data was reported between survivors and non-survivors. The studied derived hematological indices were neutrophil-to-lymphocyte ratio (NLR), derived neutrophil-to-lymphocyte ratio (dNLR, absolute neutrophil count/(white blood cells—absolute neutrophil count)), systemic inflammation index (SII, (neutrophil × platelet)/lymphocyte ratio), monocyte-to-lymphocyte ratio (MLR), and platelet-to-lymphocyte ratio (PLR). Delta values were studied for the derived hematological indices by subtracting the value at ICU admission from the value at 48 h after ICU admission. 

### 2.4. Statistical Analysis

Statistical analysis was performed with respect to the normality of data distribution tested through the Kolmogorov–Smirnov test. The collected data were categorical, ordinal, and continuous. Categorical and ordinal data were expressed as percentage or frequency and standard deviation (SD). Continuous variables were expressed as mean or median and standard deviation or interquartile range. Nonparametric tests were used to compare not normally distributed continuous data between two different groups (Mann–Whitney U test), more than two different groups (Kruskal–Wallis test), or two related groups (Wilcoxon test). After crosstabs were computed, categorical variables were compared using the Chi-square test. The discriminative power for derived hematological indices was initially assessed using receiver operating characteristic (ROC) curves. If the area under the curve (AUC) was statistically significant, the optimal cut-off value was identified using the Youden index and sensitivity, specificity, positive predictive value (PPV), and negative predictive value (NPV) were also calculated. For the given cut-off value, every hematological index or parameter was further analyzed using Cox proportional hazards (PH) regression in order to assess the independent predictive value of each parameter in uni- and multivariate analyses. The multivariate analysis included all the risk factors statistically associated in the univariate analysis with the two main events studied: (1) need for invasive mechanical ventilation and (2) death, in order to adjust the hematological indices for possible confounders. Cox PH regression results were expressed as hazard ratio (HR) at a 95% confidence interval (95% CI). The variables introduced were tested for high level multicollinearity. The IBM Statistical Package for Social Sciences (SPSS) for Windows, version 20.0, Armonk, NY, USA: IBM Corp. was used for statistical analysis.

## 3. Results

### 3.1. Baseline Characteristics of the Study Population

For this study, 272 patients met the inclusion criteria. Results will be presented with respect to the two primary end-points: need for IMV during ICU-LOS and death risk for the whole sample. Regarding gender, there were 186 males and 86 females with no significant difference across groups (*p* > 0.05). Mean age was 62.7 (±12) and was statistically significant between patients from groups 1 and 3 and patients from groups 2 and 3: 64.9 (±9.9), 65.4 (±11), and 58.5 (±12.4), respectively (*p* < 0.0001). The same observation regarding age was made between survivors and non-survivors (58.2 (±11.8) vs. 66.8 (±10.5), *p* < 0.0001). In comparison with group 3 (*p* < 0.0001), groups 1 and 2 had a higher frequency for cardiac disease (87.9% and 86.6% vs. 62.9%, *p* < 0.001), diabetes mellitus (36.4% and 47.8% vs. 26.7%, *p* = 0.004), and the Charlson Comorbidity Index values (4.45 (±2) and 4.29 (±2) vs. 2.5 (±1.77), *p* < 0.0001). Similar results were seen in survivors vs. non-survivors (Table 1). Obesity frequency was not significantly different across groups (*p* > 0.05). Patients on IMV upon admission and those requiring IMV during ICU-LOS had significantly higher mortality rates (81.7% and 76.1% vs. 13.3%, *p* < 0.0001). Also, higher mortality was observed in patients with hospital acquired infections (66.2% vs. 27.7%, *p* < 0.0001). Median value for day of invasive mechanical ventilation initiation was 5 (IQR: 3–6), while for day of death the median value was 10 (IQR: 7–15.75). Table 1 summarizes demographic and clinically relevant data between groups depending on the studied event.

### 3.2. Hematological and Biochemical Parameters Analysis at ICU Admission and 48 h

Hematological and biochemical parameters at ICU admission and 48 h are listed in Table 2. Significantly different mean values resulted between the patients from group 1 versus groups 2 and 3 regarding white blood cells (13.51 × 10^3^/mL (±6.69) vs. 10.59 × 10^3^/mL (±4.15) vs. 10.22 × 10^3^/mL (±3.8), *p* = 0.024), neutrophils (12.11 × 10^3^/mL (±6.05) vs. 9.3 × 10^3^/mL (±3.93) vs. 8.73 × 10^3^/mL (±3.58), *p* = 0.007), immature granulocytes (0.37 × 10^3^/mL (±0.47) vs. 0.17 × 10^3^/mL (±0.22) vs. 0.13 × 10^3^/mL (±0.15), *p* = 0.002), dNLR (10.12 (±5.17) vs. 8.13 (±4.23) vs. 7.14 (±3.96), *p* = 0.004), NLR (18.7 (±11.29) vs. 13.85 (±8.4) vs. 12.53 (±10.04), *p* = 0.002), and SII (4820 (±3220) vs. 3866 (±2971) vs. 3449 (±2903), *p* = 0.042). These differences were not significant between patients from group 2 (IMV during ICU-LOS) and 3 (non-IMV during ICU-LOS) at ICU admission (0 h), which at the same time had significantly different mean values for P/F ratios (117 (±51) vs. 132 (±54), *p* = 0.012). 

**Figure 1:**
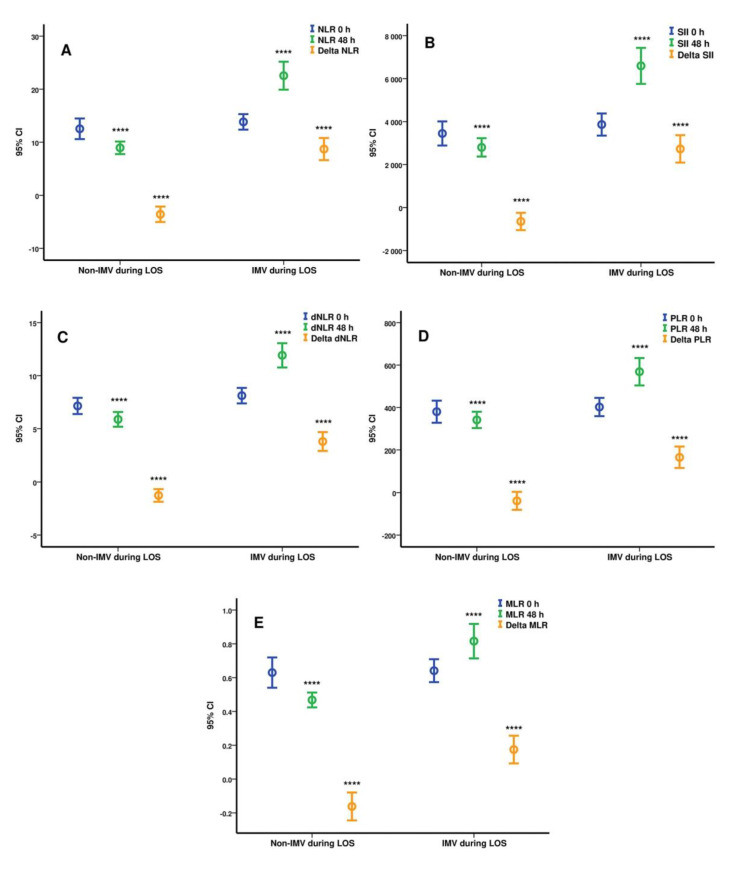
Differences for NLR (**A**), SII (**B**), dNLR (**C**), PLR (**D**), and MLR (**E**) at ICU admission and 48 h between patients requiring invasive mechanical ventilation during ICU stay versus patients on NIPPV or HFOT. Results are represented as means (circles) with 95% CI (bars), non-IMV during LOS (n = 105), IMV during LOS (n = 134); delta values = 48 h value minus ICU admission value; **** *p* < 0.0001, Statistical analysis was performed using the Mann–Whitney U Test and the statistical significance is highlighted for each parameter at 0 h, 48 h, and delta value between patients on IMV during LOS and those not on IMV during LOS. NLR = Neutrophil-to-lymphocyte ratio, SII = systemic inflammation index, dNLR = derived neutrophil-to-lymphocyte ratio, PLR = platelet-to-lymphocyte ratio, MLR = monocyte-to-lymphocyte ratio, 95% CI = 95% confidence interval, IMV = invasive mechanical ventilation, ICU = intensive care unit, LOS = length of stay, NIPPV = non-invasive positive pressure ventilation, HFOT = high-flow oxygen therapy.

Analysis of dynamic changes in hematological profile at 48 h was statistically significant between groups 2 and 3 for white blood cells (12.55 × 10^3^/mL (±4.77) vs. 9.44 × 10^3^/mL (±3.3), *p* < 0.0001), neutrophils (11.41 × 10^3^/mL (±4.63) vs. 7.8 × 10^3^/mL (±3.06), *p* < 0.0001), lymphocytes (0.66 × 10^3^/mL (±0.41) vs. 1.14 × 10^3^/mL (±0.82), *p* < 0.0001), immature granulocytes (0.26 × 10^3^/mL (±0.39) vs. 0.16 × 10^3^/mL (±0.23), *p* = 0.003), dNLR (11.89 (±6.61) vs. 5.88 (±6.61), *p* < 0.0001), NLR (22.46 (±15.18) vs. 8.95 (±6.1), *p* < 0.0001), SII (6552 (±4843) vs. 2802 (±2206), *p* < 0.0001), MLR (0.81 (±0.58) vs. 0.46 (±0.22), *p* < 0.0001), and PLR (566 (±373) vs. 342 (±199), *p* < 0.0001). Also, the delta values (difference between 48 h value and 0 h value) for the derived hematological indices were highly statistically significant between groups 2 and 3 as follows: ΔdNLR (3.76 (±5.09) vs. −1.26 (±3.09), *p* < 0.0001), ΔNLR (8.6 (±12.03) vs. −3.58 (±7.48), *p* < 0.0001), ΔSII (2686 (±3687) vs. −646 (±2080), *p* < 0.0001), ΔMLR (0.16 (±0.47) vs. −0.16 (±0.42), *p* < 0.0001), and ΔPLR (164 (±289) vs. −39 (±219), *p* < 0.0001). The statistical analysis of the hematological indices differences between groups 2 and 3 were plotted as means (circles) with 95% CI (error bars) in Figure 1.

Patients requiring IMV during ICU-LOS had higher CRP values at 48 h compared with patients from group 3 (120 mg/L (±84) vs. 88 mg/L (±73), *p* = 0.002), lower P/F ratios values (113 (±50) vs. 171 (±70), *p* < 0.0001) and lower D-dimers value, but with higher sum of ranks (2098 ng/mL (±4800) vs. 2432 ng/mL (±10,031), 17,951.5 vs. 10,489.5, *p* < 0.0001). Bivariate analysis using the Spearman correlation showed a strong significant positive correlation between 48 h D-dimers, CRP values, and dNLR, NLR, SII (*p* < 0.001), and PLR (*p* = 0.001), but not MLR (*p* > 0.05), and a strong negative correlation between P/F ratio and dNLR, NLR, SII, MLR, and PLR (*p* < 0.001). 

At ICU admission, non-survivors had significantly lower lymphocytes values (0.99 × 10^3^/mL (±0.87) vs. 0.77 × 10^3^/mL (±0.4), *p* = 0.003) and higher dNLR (7.13 (±3.9) vs. 8.77 (±4.58), *p* = 0.002), NLR (12.34 (±9.54) vs. 15.4 (±9.43), *p* = 0.001), SII (3440 (±2868) vs. 4169 (±3073), *p* = 0.018), MLR (0.61 (±0.44) vs. 0.69 (±0.4), *p* = 0.017), and PLR (371 (±253) vs. 419 (±263), *p* = 0.04) values.

**Figure 2:**
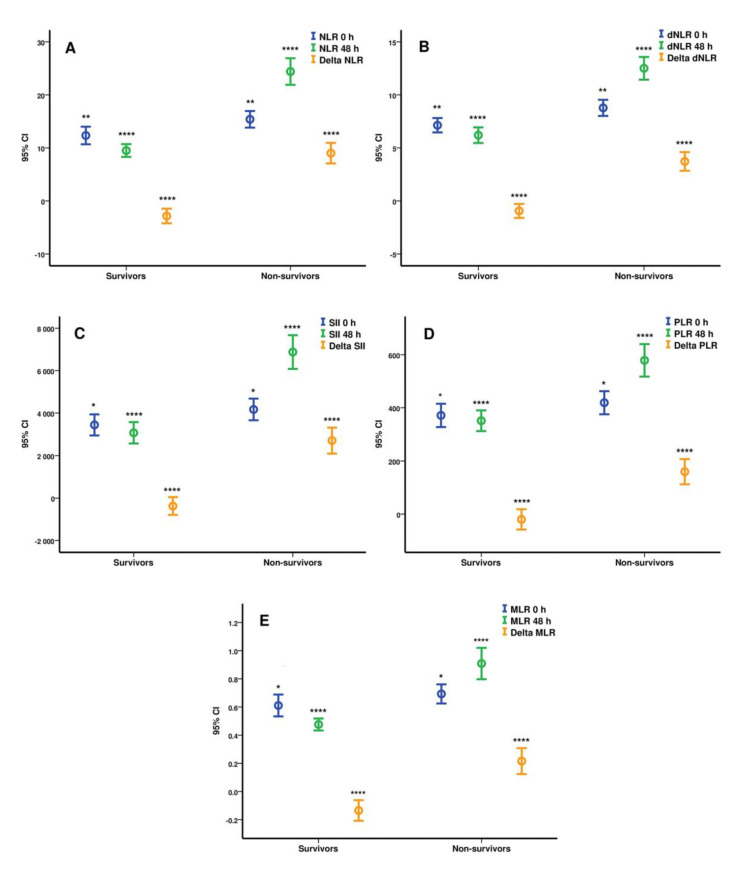
Differences for NLR (**A**), dNLR (**B**), SII (**C**), PLR (**D**), and MLR (**E**) at ICU admission and 48 h between survivors and non-survivors. Results are represented as means (circles) with 95% CI (bars), survivors (n = 130), non-survivors (n = 142), delta values = 48 h value minus ICU admission value; * *p* < 0.05, ** *p* < 0.01, **** *p* < 0.0001. Statistical analysis was performed using the Mann–Whitney U Test and the statistical significance is highlighted for each parameter at 0 h, 48 h, and delta value between survivors and non-survivors. NLR = neutrophil-to-lymphocyte ratio, dNLR = derived neutrophil-to-lymphocyte ratio, SII = systemic inflammation index, PLR = platelet-to-lymphocyte ratio, MLR = monocyte-to-lymphocyte ratio, 95% CI = 95% confidence interval, ICU = intensive care unit, LOS = length of stay.

Further, at 48 h non-survivors had higher WBC (9.8 × 10^3^/mL (±3.73) vs. 13.24 × 10^3^/mL (±5.61), *p* < 0.0001), neutrophils (8.15 × 10^3^/mL (±3.56) vs. 12.12 × 10^3^/mL (±5.3), *p* < 0.0001), immature granulocytes (0.2 × 10^3^/mL (±0.28) vs. 0.33 × 10^3^/mL (±0.57), *p* = 0.004), dNLR (−0.93 (±3.79) vs. 3.72 (±5.3), *p* < 0.0001), NLR (−2.83 (±7.92) vs. 9 (±11.71), *p* < 0.0001), SII (−372 (±2416) vs. 2706 (±3678), *p* < 0.0001), MLR (−0.13 (±0.42) vs. 0.21 (±0.55), *p* < 0.0001), PLR (−20 (±221) vs. 156 (±287), *p* < 0.0001), and lower lymphocytes (1.14 × 10^3^/mL (±0.8) vs. 0.61 × 10^3^/mL (±0.36), *p* < 0.0001) and platelets (311 × 10^3^/mL (±104) vs. 288 × 10^3^/mL (±123), *p* = 0.034) mean value. The statistical analysis of the hematological indices differences between groups 2 and 3 were plotted as means (circles) with 95% CI (error bars) in Figure 2.

Finally, non-survivors had higher CRP (94 mg/L (±79) vs. 121 mg/L (±85), *p* = 0.004), D-dimers (2186 ng/mL (±9046) vs. 2329 (±4955), *p* < 0.0001), and lower P/F ratio (162 (±70) vs. 117 (±52), *p* < 0.0001) values at 48 h.

### 3.3. Prediction Analysis and Cut-Off Values Identification Using ROC Curves for the Studied Hematological Indices

Receiver operating characteristic (ROC) curves were plotted for hematological indices at ICU admission (0 h), 48 h, and delta values in order to test their discriminative power for the need of IMV and death prediction. Area under the curve (AUC) values for each parameter are listed in Table 3. For the need of IMV prediction, delta values performed better than 48 h values, although the AUC was increased marginally (AUC NLR 48 h = 0.840 vs. AUC ΔNLR = 0.876, AUC dNLR 48 h = 0.812 vs. AUC ΔdNLR = 0.826, AUC SII = 0.786 vs. AUC ΔSII = 0.834, AUC MLR = 0.709 vs. AUC ΔMLR = 0.713, and AUC PLR = 0.730 vs. AUC ΔPLR = 0.774). For each parameter, optimal cut-off values with their sensitivity (Sn) and specificity (Sp) were identified for need of IMV as follows: ΔNLR value >2, Sn = 79.5%, Sp = 91.4%; ΔdNLR > 1: Sn = 70.5%, Sp = 84.8%; ΔSII > 340: Sn = 79.5%, Sp = 80%; ΔMLR > 0.1: Sb = 53.8%, Sp = 81.9%; and ΔPLR > 50: Sn = 68.2%, Sp = 79%.

On the other hand, for death prediction, AUCs at 48 h had better discriminative power compared with delta values, but their difference was also marginal: ΔNLR = 0.846 vs. NLR 48 h = 0.867, ΔSII = 0.793 vs. SII 48 h = 0.796, ΔdNLR = 0.791 vs. dNLR 48 h = 0.831, ΔPLR = 0.740 vs. PLR 48 h = 0.742, and ΔMLR = 0.700 vs. MLR 48 h = 0.747. In a similar fashion, optimal cut-off values were identified for death prediction: NLR 48 h > 11, Sn = 86.6%, Sp = 72.3%, SII 48 h > 3700, Sn = 71.8%, Sp = 70.8%, dNLR 48 h > 6.93, Sn = 80.3%, Sp = 70%, PLR 48 h > 300, Sn = 82.4%, Sp = 49.2%, MLR 48 h, Sn = 60%, and Sp = 80.8%. Positive predictive values (PPVs) and negative predictive values (NPVs) for all the aforementioned cut-off values are listed in Table 3.

### 3.4. Independent Predictive Value of Hematological Indices after Multivariate Analysis

For the identified cut-off values, each hematological index was further analyzed using Cox proportional hazards (PH) regression. In order to test each hematological index as a potential independent risk factor, multivariate analysis was performed for adjustment with possible confounders (variables that in the current analysis were significantly associated with the studied events).

For the outcome (1) need for invasive mechanical ventilation, the hematological parameters were adjusted for age > 60 years, CRP level at 48 h, need for NIPPV (non-invasive positive pressure ventilation), severe hypoxemia at 48 h (P/F ratio < 100), diabetes mellitus, and the Charlson Comorbidity Index, while for the outcome (2) death, the hematological indices were adjusted for age > 60 years, the Charlson Comorbidity Index, SOFA score value at 48 h, healthcare associated infections (HAIs), CRP value at 48 h, severe hypoxemia at 48 h (P/F ratio < 125), tocilizumab therapy, and the need for higher respiratory support (need for NIPPV or IPPV versus HFOT during LOS). 

The complete Cox PH regression models for IMV requirement and death are listed in Table 4 and Table 5, respectively. Corticosteroid therapy was not significantly associated with the aforementioned events in the univariate analysis; therefore, hematological indices were adjusted for the death event only for immunotherapy with tocilizumab.

Regarding the need for IMV the adjusted hazard ratio for each hematological index after multivariate analysis is as follows: ΔNLR > 2: HR = 5.05 (95% CI: 3.06–8.33, *p* < 0.0001), ΔSII > 340: HR = 3.56 (95% CI: 2.21–5.74, *p* < 0.0001), ΔdNLR > 1: HR = 2.61 (95% CI: 1.7–4.01, *p* < 0.0001), ΔPLR > 50: HR = 1.95 (95% CI: 1.29–2.93, *p* = 0.001), and ΔMLR > 0.1: HR = 1.73 (95% CI: 1.19–2.51, *p* = 0.004). 

**Figure 3:**
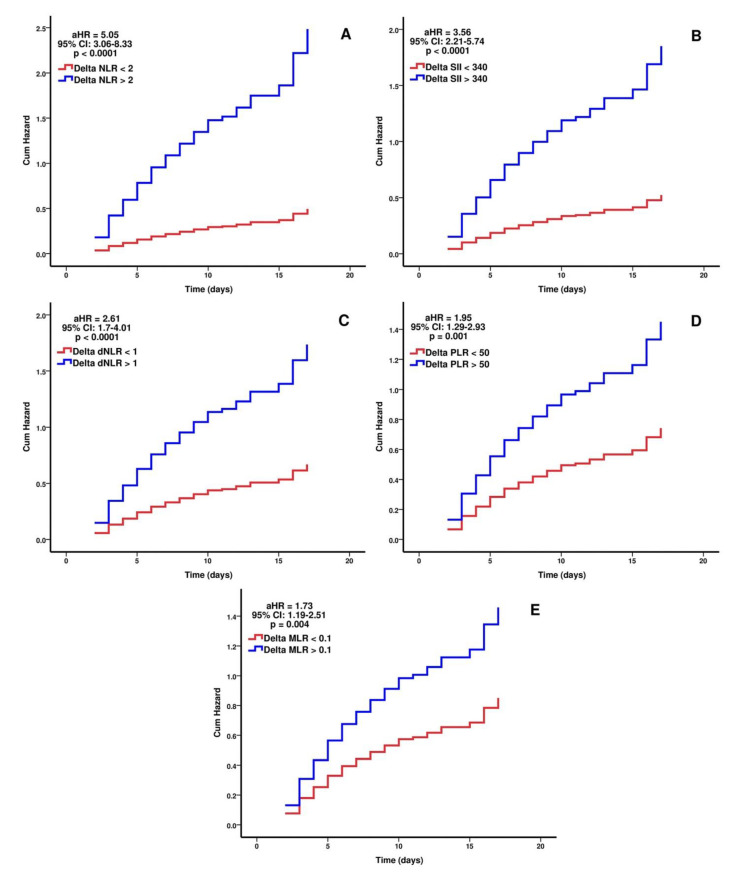
The independent predictive value of ΔNLR (**A**), ΔSII (**B**), ΔdNLR (**C**), ΔPLR (**D**), and ΔMLR (**E**) for invasive mechanical ventilation need in patients with COVID-19 not on IMV at ICU admission. Cumulative hazard curves were plotted for each independent predictor after multivariate analysis and results are expressed as the adjusted hazard ratio. Multivariate analysis was performed using Cox proportional hazards regression models. Non-IMV during LOS (n = 105), IMV during LOS (n = 134); ΔNLR = delta neutrophil-to-lymphocyte ratio, ΔSII = delta systemic inflammation index, ΔdNLR = delta derived neutrophil-to-lymphocyte ratio, ΔPLR = delta platelet-to-lymphocyte ratio, ΔMLR = delta monocyte-to-lymphocyte ratio, 95% CI = 95% confidence interval, IMV = invasive mechanical ventilation, ICU = intensive care unit, LOS = length of stay, aHR = adjusted hazard ratio.

In all the Cox PH regression models, NIPPV requirement and a P/F ratio < 100 at 48 h were also independently associated with the need for invasive mechanical ventilation, with hazard ratios between 1.89 and 2.04 (95% CI, 1.28–3, *p* < 0.01) for NIPPV and HR between 1.52 and 2.17 (95% CI, 1.05–3.16, *p* < 0.01) for P/F ratio < 100 at 48 h, respectively. In the regression models for ΔSII and ΔMLR, CRP value at 48 h was independently associated with the need for mechanical ventilation (HR = 1.003, 95% CI: 1.001–1.005, *p* < 0.05). All the regression models are listed in Table 4, while the adjusted hazard ratios for the studied hematological indices were plotted in Figure 3. 

For the second outcome studied, death, the adjusted HR for hematological indices at 48 h after multivariate analysis were NLR > 11: HR = 2.25 (95% CI: 1.31–3.86, *p* = 0.003), SII > 3700: HR = 1.68 (95% CI: 1.13–2.49, *p* = 0.01), dNLR > 6.93: HR = 1.89 (95% CI: 1.2–2.98, *p* = 0.005), PLR > 300: HR = 1.66 (95% CI: 1.04–2.64, *p* = 0.025), and MLR > 0.64: HR = 1.49 (95% CI 1.003–2.2, *p* = 0.048). 

**Figure 4:**
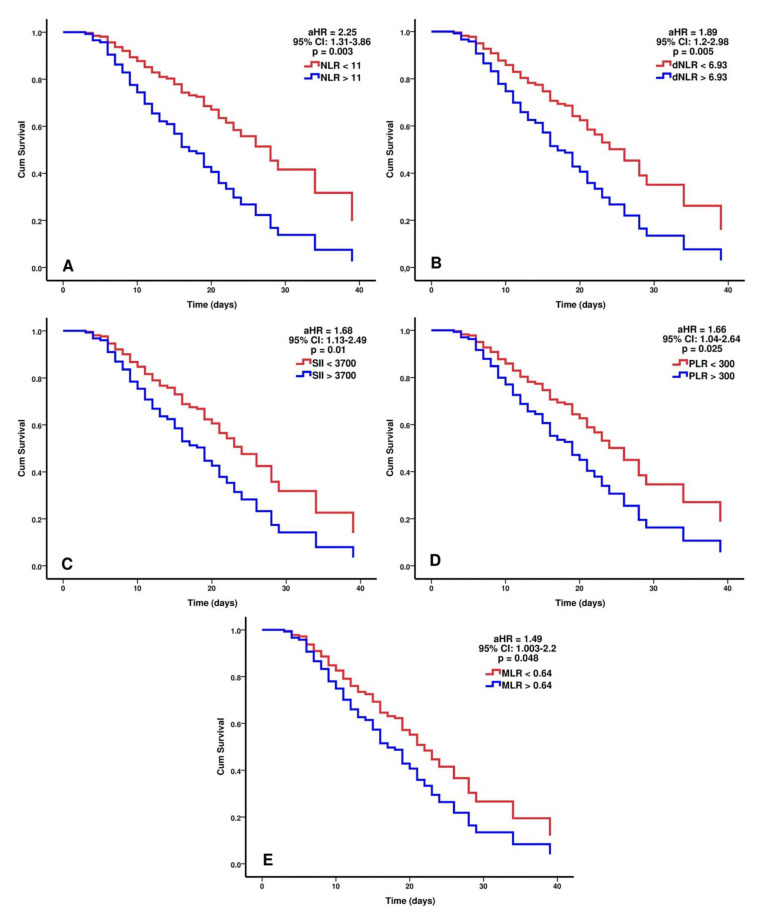
The independent predictive value of NLR (**A**), dNLR (**B**), SII (**C**), PLR (**D**), and MLR (**E**) for death in critically ill COVID-19 patients. Survival curves were plotted for each independent predictor after multivariate analysis and results are expressed as the adjusted hazard ratio. Multivariate analysis was performed using Cox proportional hazards regression models. Survivors (n = 130), non-survivors (n = 142); NLR = neutrophil to lymphocyte ratio, dNLR = derived neutrophil-to-lymphocyte ratio, SII = systemic inflammation index, PLR = platelet-to-lymphocyte ratio, MLR = monocyte-to-lymphocyte ratio, 95% CI = 95% confidence interval, LOS = length of stay, aHR = adjusted hazard ratio.

In all the regression models, HAIs and higher respiratory support needed were strongly associated with death and had higher hazard ratios in contrast with hematological indices, ranging between 2.19–2.31 (95% CI: 1.48–3.4, *p* < 0.0001) for HAIs and between 3.48–3.76 (95% CI: 2.30–5.62, *p* < 0.0001) for higher respiratory support. P/F ratio < 125 at 48 h is another risk factor significantly associated with death in all the regression models with HR between 1.88–2.1 (95% CI: 1.28–3.09, *p* < 0.01). Immunotherapy with tocilizumab was independently associated with survival in one regression model (HR = 0.52, 95% CI: 0.28–0.97, *p* = 0.041). All the regression models are listed in Table 5, while the adjusted hazard ratios for the studied hematological indices were plotted in Figure 4. 

We also find it important to report the possible effect of associated diseases, corticosteroid treatment, and immunotherapy on these hematological indices. Data about precise corticosteroid treatment were available only for 165 out of 272 patients. Thirty-eight patients received a 6 mg dexamethasone regimen, 70 patients were on high dose dexamethasone therapy (16 mg), and 50 patients received methylprednisolone. 

No significant difference was observed at ICU admission for NLR, dNLR, SII, PLR, and MLR between patients with or without obesity (*p* > 0.05), diabetes mellitus (*p* > 0.05), cardiac disease (*p* > 0.05), or chronic kidney disease (*p* > 0.05). Also, at 48 h, no significant difference was observed between patients on low-dose dexamethasone (*p* > 0.05) or high-dose dexamethasone (*p* > 0.05), but differences were significant for NLR, dNLR, and SII (*p* < 0.05) for those on methylprednisolone therapy. In these patients (i.e., those on methylprednisolone therapy) the same statistical correlations were observed at ICU admission (before methylprednisolone initiation) for the mentioned hematological indices. 

Patients receiving immunotherapy with tocilizumab had significantly lower values at 48 h for NLR (9.66 vs. 18.36, *p* < 0.001), dNLR (7.04 vs. 9.83, *p* < 0.001), SII (2734 vs. 5387, *p* < 0.001), PLR (329 vs. 490, *p* < 0.001), and MLR (0.34 vs. 0.75, *p* < 0.001) and had higher absolute lymphocyte count (1.09 vs. 0.83, *p* < 0.001). As mentioned previously, in univariate analysis, only treatment with tocilizumab was significantly (and negatively) associated with death; it was therefore introduced as a variable in the Cox PH regression model. Treatment with dexamethasone (regardless of dose) or methylprednisolone were not significantly associated with the studied events and were not introduced in the regression models.

## 4. Discussion

Our study describes the importance of dynamic hematological changes in 272 COVID-19 patients admitted to the ICU and their predictive value for invasive mechanical ventilation need and death. We demonstrated that dynamic changes in hematological indices are strongly correlated with disease progression and severity, systemic inflammation, and death. Furthermore, NLR had the best independent predictive values for the studied events (invasive mechanical ventilation need and death) followed by SII and dNLR. To our knowledge, this is the first study that reports the independent risk value of dynamic hematological values at 48 h and delta values for IMV need and death in critically ill patients with COVID-19.

Age and gender distribution was similar to the published data [16]. As reported, cardiac disease [16,17], diabetes mellitus [16,17], and a higher Charlson Comorbidity Index [15] were associated with disease severity [15,16,17], need for IMV [15,16,17], and death [15,16]. Interestingly, there were no significant differences regarding these two outcomes in obese patients, although several studies found it as a risk factor [17,18]. Patients requiring IMV and non-survivors had higher 48 h SOFA scores compared with patients not on IMV and survivors. 

Hematological disturbances at ICU admission were in line with those reported in other studies [8,9,10,11,12,13,14,15,19]. Lymphopenia, neutrophilia, and leukocytosis are frequently observed in patients with COVID-19 [8,9,10,11,12,19]. Bolondi et al. documented that lymphopenia reaches a nadir in day 2 of ICU stay, but the CD4/CD8 ratio is conserved [20]. Huang et al. [11] made similar observations in a meta-analysis, concluding that all lymphocyte subsets are decreased in COVID-19 critical patients. The possible mechanisms involved include direct lymphocyte lysis through virus binding to ACE2 receptors present on their surface, lymphocyte apoptosis secondary to cytokine storm [9], and T cell exhaustion [5]. Moreover, lymphopenia is negatively correlated with viral load [21]. We found similar results in this study. More precisely, non-survivors and patients requiring IMV had significantly lower lymphocytes at 48 h (or day 2) compared with survivors or patients not on IMV and compared with ICU admission values.

Recently, Metzemaekers et al. studied the kinetics of peripheral blood neutrophils in severe COVID-19. Not only did patients with severe COVID-19 have higher neutrophil count compared with non-severe patients, but they also had an increased number of immature and activated neutrophils. A possible mechanism for neutrophil activation may be attributable to CXCR2 and C5aR down-regulation. In addition, in patients with severe COVID-19, increased concentrations of neutrophil elastase, gelatinolytic activity, and TIMP-1/MMP-9 complexes were found [22]. In our study, patients requiring IMV upon ICU admission had higher mean values for immature granulocytes compared with patients not on IPPV. Moreover, patients requiring IMV and non-survivors had significantly higher immature granulocytes values at 48 h. The aforementioned mechanisms can, in part, explain our findings.

NLR is the most studied hematological index in COVID-19 patients and higher values were correlated with disease severity and progression [12,13,23,24,25], death [13,14,22,24,26], and treatment response [27]. Our observations are in agreement for NLR but also for PLR, MLR, SII, and dNLR and are correlated with results from other studies [11,12,13,14,15,23,24,25,26,28,29,30,31]. In our study, dynamic changes of these hematological indices had stronger correlations and better predictive value for progression to IMV in non-intubated patients and death compared with ICU admission values. A few studies concluded that dynamic changes of NLR, PLR, and MLR [10,29,30,31] were useful for discriminating between severe and non-severe cases in time. Thus, using only the admission values to predict disease worsening and death may not be as reliable as the dynamic hematological changes given the complex and unpredictable evolution of critical illness in COVID-19 patients. In our study, this was highlighted by the change in the discriminative power (value of AUC) of these indices over time. AUC values for the derived hematological indices, even the ICU admission values, are roughly in line with the AUC reported in other studies [12,15,23,24,25,26,27,28,29,30,31,32], but different cut-off values were taken. For example, the cut-off value range for NLR as a predictor for disease severity or death is very wide—from 3.3 to 15.2 [12,15,24,27,32]—making it difficult to establish a final cut-off value. We must take into account that in this study only patients with COVID-19 admitted to the ICU were introduced, while values reported in many studies are from all patients hospitalized with COVID-19 in a given period. Lastly, based on a retrospective analysis of a cohort of 12,862 patients, Cai et al. suggested that corticosteroid therapy should be initiated in COVID-19 patients with an NLR > 6,11, being associated with reduced mortality [27]. 

Of all the studied hematological indices, delta NLR values > 2 at 48 h were the best independent predictor for IMV need, followed by delta SII > 340 and delta dNLR > 1 with HRs of 5.05 (95% CI: 3.06–8.33, *p* < 0.001), 3.56 (95% CI: 2.12–5.74, *p* < 0.001), and 2.61 (95% CI: 1.7–4.01, *p* < 0.001), respectively. PLR and MLR were also found to be independent predictors for IMV need, but with lower hazard ratio values. Lastly, a P/F ratio < 100 at 48 h and the need for NIPPV were independent risk factors associated with IMV need in our study and are known risk factors in other studies [16]. 

Even though these indices performed very well for IMV prediction, they were independently associated with death, but to a lesser extent, 48 h values of NLR > 11, SII > 3700, and dNLR > 6.93 having HR = 2.25 (95% CI: 1.31–3.86, *p* = 0.003), HR = 1.68 (95% CI: 1.13–2.49, *p* = 0.01), and HR = 1.89 (95% CI: 1.2–2.98, *p* = 0.005), respectively. Moreover, in our study, death was independently predicted by a higher respiratory support needed (NIPPV or IPPV versus HFO) (HR = 3.48–3.76, 95% CI: 2.30–5.62, *p* < 0.0001), sepsis secondary to hospital acquired infections (HR = 2.19–2.31, 95% CI: 1.48–3.4, *p* < 0.0001), and hypoxemia severity (HR = 1.88–2.1, 95% CI: 1.28–3.09, *p* < 0.01). These are known independent risk factors for death [16]. Immunotherapy with tocilizumab in the first 36 h (early initiation) was an independent predictor for survival with an HR = 0.52 (95% CI: 0.28–0.97, *p* = 0.041) and a similar observation was made in another study [33]. NLR was found to have independent predictive value for death in a meta-analysis with a reported relative risk (RR) of 2.74 (95% CI: 0.98–7.66) regardless of the cut-off values used [24], and this result is in line with the hazard ratio we described earlier for an NLR > 11. SII was also reported as an independent risk factor for in-hospital mortality [15]. 

All of these hematological indices were adjusted for the possible confounders, but some aspects should also be considered. All of these hematological indices can be or are increased by associated diseases like diabetes mellitus [34], cardiac failure [35], chronic kidney disease [36], and anti-inflammatory medication (corticosteroids) [37]. In our study, at admission, there were no differences for the studied indices between patients with these associated diseases and those without these conditions, or between patients on low-dose and patients on high-dose dexamethasone at 48 h. Although in the case of methylprednisolone therapy at 48 h there were significant differences for NLR, dNLR, and SII, as we reported in the results section, these differences were also significant at ICU admission (before methylprednisolone initiation) and may have represented only a trend maintenance over time. Moreover, corticosteroids do not seem to change neutrophil phenotype and polarization in severe COVID-19 patients [22]. Even with these results, we must make two observations: (1) unfortunately, data about corticosteroid therapy administered previously to ICU admission was not available and (2) data about precise corticosteroid dosage was available only for 165 patients out of 272. 

Furthermore, immunotherapy with tocilizumab appeared to change the values of these indices. Patients treated with it had lower NLR, dNLR, SII, PLR, and MLR at 48 h and delta values and higher absolute lymphocyte count values and survival rates. These findings are in agreement with results reported by other researchers for COVID-19 patients that received tocilizumab [38]. The positive effect of tocilizumab in other diseases seemed to be represented by a drop in NLR and neutrophil count [39,40]. The predictive value of the hematological parameters was adjusted for these factors accordingly and we considered it of utter importance to reduce the possible bias. Moreover, this is the first study to adjust or to analyze the predictive value of these hematological parameters for immunotherapy in COVID-19 patients. 

Lastly, in order to reduce the possible bias and as a major strength of our study, patients with end-stage organ disease, patients with ongoing chemo-, radio-, or immunotherapy, patients aged >85 years old, and those with suspected or confirmed bacterial infections at ICU admission were excluded.

However, our study has some limitations. Firstly, the retrospective nature of the study, even with good inclusion/exclusion criteria, is prone to bias. Secondly, the sample is not large enough to make conclusions for the general population, even if the patients were included from ICUs from two different tertiary centers. Furthermore, precise data about length and dosage of corticosteroid therapy prior to ICU admission were not available. This is important given the possible effects of corticosteroid therapy on lymphocyte count and subsequently on hematological indices values. Lastly, as mentioned earlier, even if we adjusted all of these hematological indices for the possible confounders, the homeostasis of critical care patients is profoundly disturbed and the number of different blood cells may be changed by these alterations. Perhaps using these ratios is more useful and solid than just the absolute counts of blood cells alone, given the complex immunological mechanisms of viral sepsis, and more constant regardless of the effect on their count by critical illness. 

## 5. Conclusions

This study is the first to describe the independent and better predictive value of the dynamic hematological indices at 48 h (and delta values) after ICU admission in patients with COVID-19. By far, from all hematological indices, NLR is the best independent predictor for invasive mechanical ventilation need and death, followed by SII and dNLR. These indices are strongly correlated with respiratory failure severity and systemic inflammation and can be used as biomarkers for disease severity and progression, triage, and outcome. More studies are needed to identify their optimal cut-off values. Nonetheless, irrespective of their independent predictive value, all of these hematological indices should be integrated for better clarity in the “bigger picture” with other laboratory tests, radio-imagistic findings, and clinical examination. Even with these results, we must take into consideration that mortality was also best predicted by the presence of severe respiratory failure requiring invasive mechanical ventilation and sepsis secondary to hospital acquired infections.

## Figures and Tables

**Table 1:** Demographic and clinically relevant data.

	Total Sample N = 272	Group 1 N = 33	Group 2 N = 134	Group 3 N = 105	*p* Value	Survivors N = 130	Non-Survivors N = 142	*p* Value
**Age ^¶^**	62.7 ± 12	64.9 ± 9.9	65.4 ± 11	58.5 ± 12.4	<0.0001 *	58.2 ± 11.8	66.8 ± 10.5	<0.0001 *
**Gender F/M%**	31.6/68.4	33.3/66.7	35.1/64.9	26.7/73.3	0.372 **	28.5/71.5	34.5/65.5	0.284 **
**Obesity ^⸕^**	30.9/69.1	30.3/69.7	26.9/73.1	36.2/63.8	0.301 **	33.1/66.9	28.9/71.1	0.454 **
**CCI ^¶^**	3.61 ± 2.13	4.45 ± 2	4.29 ± 2	2.5 ± 1.77	<0.0001 *	2.53 ± 1.7	4.6 ± 2	<0.0001 ***
**SOFA Score 0 h ^¶^**	4 ± 1.7	6.88 ± 2.26	3.72 ± 1.23	3.45 ± 1	<0.0001 *	3.62 ± 1.36	4.34 ± 1.9	0.001 ***
**SOFA Score 48 h ^¶^**	5.1 ± 2.9	8.55 ± 2.91	6.24 ± 2.21	4 ± 1.7	<0.0001 *	3.25 (±1.84)	6.73 ± 2.65	<0.0001 ***
**Cardiac disease ^⸕^**	22.4/77.6	12.1/87.9	13.4/86.6	37.1/62.9	<0.001 **	33.8/66.2	12/88	<0.001 **
**Diabetes mellitus ^⸕^**	61.8/38.2	63.6/36.4	52.2/47.8	73.3/26.7	0.004 **	71.5/28.5	52.8/47.2	0.002 **
**Respiratory disease^⸕^**	83.46/16.54	63.6/36.4	81.35/18.65	92.24/7.76	0.067 **	90/10	77.47/22.53	0.082 **
**CKD ^⸕^**	90.1/9.9	84.8/15.2	88.8/11.2	93.3/6.7	0.287 **	92.3/7.7	88/12	0.238 **
**Liver disease ^⸕^**	90/10	78.79/21.21	89.56/10.44	94.3/5.7	0.082 **	90/10	88.02/11.98	0.104 **
**Cancer ^⸕^**	96/4	91/9	97/3	96.2/3.8	0.13 **	96.15/3.85	95.78/4.22	0.22 **
**Tocilizumab ^⸕^**	87.5/12.5	87.9/12.1	89.6/10.4	84.8/15.2	0.538 **	83.1/16.9	91.5/8.5	0.035 **
**Anakinra ^⸕^**	91.5/8.5	100/0	88.8/11.2	92.4/7.6	0.109 **	91.5/8.5	91.5/8.5	0.997 **
**Remdesivir ^⸕^**	63.6/36.4	69.7/30.3	55.2/44.8	72.4/27.6	0.018 **	70/30	57.7/42.3	0.036 **
**HAIs ^⸕^**	52.2/47.8	45.5/54.5	29.1/70.9	83.8/16.2	<0.0001 **	72.3/27.7	33.8/66.2	<0.0001 ***
**ICU LOS ^¶^**	12.97 ± 7	10.6 ± 6	14.14 ± 8.6	12.25 ± 4.3	0.044 *	14.2 ± 7.1	11.9 ± 6.8	<0.001 **
**ICU mortality%**	52.2	81.7	76.1	13.3	<0.0001 **			

Group 1 = IMV upon ICU admission, group 2 = IMV during length of stay, group 3 = non-IMV during length of stay; IMV = invasive mechanical ventilation, CCI = Charlson Comorbidity Index, SOFA = Sequential Organ Failure Assessment, CKD = chronic kidney disease, HAIs = hospital acquired infections, ICU = intensive care unit, LOS = length of stay; ¶ = values expressed as means and standard deviations, ⸕ = variable expressed as percentage (No/Yes %); * = Kruskal–Wallis H Test, ** = Chi-square test, *** = Mann–Whitney U Test; statistical significance considered for a *p* value < 0.05.

**Table 2:** Hematological and biochemical values at ICU admission and 48 h.

Values at ICU Admission (Mean ± SD)	Total Sample N = 272	Group 1 N = 33	Group 2 N = 134	Group 3 N = 105	*p* Value	Survivors N = 130	Non-Survivors N = 142	*p* Value
**White blood cells (×10^3^/μL)**	10.8 ± 4.51	13.51 ± 6.69	10.59 ± 4.15	10.22 ± 3.8	0.024 *	10.42 ± 4.08	11.16 ± 4.85	0.380 **
**Neutrophils (×10^3^/μL)**	9.42 ± 4.23	12.11 ± 6.05	9.3 ± 3.93	8.73 ± 3.58	0.007 *	8.92 ± 3.85	9.88 ± 4.51	0.113 **
**Lymphocytes (×10^3^/μL)**	0.87 ± 0.68	0.82 ± 0.56	0.8 ± 0.37	0.99 ± 0.94	0.225 *	0.99 ± 0.87	0.77 ± 0.4	0.003 **
**Monocytes (×10^3^/μL)**	0.48 ± 0.29	0.58 ± 0.44	0.46 ± 0.27	0.48 ± 0.26	0.425 *	0.48 ± 0.26	0.49 ± 0.32	0.672 **
**Platelets (×10^3^/μL)**	271 ± 106	256 ± 94	271 ± 112	275 ± 101	0.694 *	277 ± 102	265 ± 110	0.207 **
**dNLR**	7.99 ± 4.34	10.12 ± 5.17	8.13 ± 4.23	7.14 ± 3.96	0.004 *	7.13 ± 3.9	8.77 ± 4.58	0.002 **
**NLR**	13.93 ± 9.59	18.7 ± 11.29	13.85 ± 8.4	12.53 ± 10.04	0.002 *	12.34 ± 9.54	15.4 ± 9.43	0.001 **
**SII**	3821 ± 2994	4820 ± 3220	3866 ± 2971	3449 ± 2903	0.042 *	3440 ± 2868	4169 ± 3073	0.018 **
**MLR**	0.65 ± 0.42	0.77 ± 0.44	0.64 ± 0.39	0.62 ± 0.46	0.114 *	0.61 ± 0.44	0.69 ± 0.4	0.017 **
**PLR**	396 ± 259	426 ± 275	402 ± 248	380 ± 269	0.332 *	371 ± 253	419 ± 263	0.04 **
**C-reactive protein (mg/L)**	143 ± 90	163 ± 90	147 ± 91	133 ± 87	0.169 *	137 ± 90	150 ± 89	0.166 **
**D-dimers (ng/mL)**	2910 ± 8728	3930 ± 8272	2645 ± 7985	2924 ± 9770	<0.001 *	2588 ± 8853	3207 ± 8632	<0.001 **
**P/F ratio**	125 ± 54	134 ± 64	117 ± 51	132 ± 54	0.039 *	132 ± 56	117 ± 52	0.011 **
**Values at 48 h (Mean ± SD)**								
**White blood cells (×10^3^/μL)**	11.59 ± 5.09	14.53 ± 7.86	12.55 ± 4.77	9.44 ± 3.3	<0.0001 *	9.8 ± 3.73	13.24 ± 5.61	<0.0001 **
**Neutrophils (×10^3^/μL)**	10.22 ± 4.95	13.12 ± 7.39	11.41 ± 4.63	7.8 ± 3.06	<0.0001 *	8.15 ± 3.56	12.12 ± 5.3	<0.0001 **
**Lymphocytes (×10^3^/μL)**	0.86 ± 0.66	0.8 ± 0.62	0.66 ± 0.41	1.14 ± 0.82	<0.0001 *	1.14 ± 0.8	0.61 ± 0.36	<0.0001 **
**Monocytes (×10^3^/μL)**	0.47 ± 0.28	0.57 ± 0.35	0.45 ± 0.27	0.48 ± 0.28	0.085	0.48 ± 0.27	0.47 ± 0.29	0.555 **
**Platelets (×10^3^/μL)**	299 ± 115	277 ± 141	296 ± 114	309 ± 106	0.190	311 ± 104	288 ± 123	0.034 **
**dNLR**	9.49 ± 6.33	11.21 ± 6.79	11.89 ± 6.61	5.88 ± 6.61	<0.0001 *	6.2 ± 4.24	12.5 ± 6.45	<0.0001 **
**NLR**	17.28 ± 14.05	22.76 ± 14.92	22.46 ± 15.18	8.95 ± 6.1	<0.0001 *	9.5 ± 6.94	24.4 ± 15.12	<0.0001 **
**SII**	5055 ± 4417	6148 ± 4966	6552 ± 4843	2802 ± 2206	<0.0001 *	3068 ± 2893	6875 ± 4782	<0.0001 **
**MLR**	0.7 ± 0.55	0.99 ± 0.83	0.81 ± 0.58	0.46 ± 0.22	<0.0001 *	0.47 ± 0.24	0.9 ± 0.67	<0.0001 **
**PLR**	470 ± 329	489 ± 342	566 ± 373	342 ± 199	<0.0001 *	351 ± 226	579 ± 369	<0.0001 **
**Delta dNLR**	1.49 ± 5.19	1.09 ± 6.53	3.76 ± 5.09	−1.26 ± 3.09	<0.0001 *	−0.93 ± 3.79	3.72 ± 5.3	<0.0001 **
**Delta NLR**	3.34 ± 11.68	4.05 ± 9.87	8.6 ± 12.03	−3.58 ± 7.48	<0.0001 *	−2.83 ± 7.92	9 ± 11.71	<0.0001 **
**Delta SII**	1234 ± 3491	1327 ± 3492	2686 ± 3687	−646 ± 2080	<0.0001 *	−372 ± 2416	2706 ± 3678	<0.0001 **
**Delta MLR**	0.04 ± 0.52	0.22 ± 0.75	0.16 ± 0.47	−0.16 ± 0.42	<0.0001 *	−0.13 ± 0.42	0.21 ± 0.55	<0.0001 **
**Delta PLR**	74 ± 272	64 ± 225	164 ± 289	−39 ± 219	<0.0001 *	−20 ± 221	160 ± 287	<0.0001 **
**C-reactive protein (mg/L)**	108 ± 83	125 ± 96	120 ± 84	88 ± 73	0.004 *	94 ± 79	121 ± 85	0.004 **
**D-dimers (ng/mL)**	2261 ± 7200	2368 ± 3883	2098 ± 4800	2432 ± 10031	<0.0001 *	2186 ± 9046	2329 ± 4955	<0.0001 **
**P/F ratio**	139 ± 65	143 ± 55	113 ± 50	171 ± 70	<0.0001 *	162 ± 70	117 ± 52	<0.0001 **

Group 1 = intubated upon ICU admission, group 2 = intubated during length of stay, group 3 = not intubated during length of stay, dNLR = derived neutrophil-to-lymphocyte ratio, NLR = neutrophil-to-lymphocyte ratio, SII = systemic inflammation index, MLR = monocyte-to-lymphocyte ratio, PLR = platelet-to-lymphocyte ratio; delta values = value at 48 h minus ICU admission value; ICU = intensive care unit; * Kruskal–Wallis H Test, ** Mann–Whitney U Test; statistical significance considered for a *p* value < 0,05; values listed as means and standard deviations.

**Table 3:** Invasive mechanical ventilation need and death prediction for NLR, SII, dNLR, PLR, and MLR at 0 h and 48 h.

	Need for IMV Prediction							Death Prediction						
	AUC			95% CI		*p* Value		AUC			95% CI		*p* Value	
**NLR 0 h**	0.573			0.500–0.647		0.052		0.621			0.554–0.687		0.001	
**SII 0 h**	0.548			0.475–0.622		0.201		0.583			0.516–0.651		0.018	
**dNLR 0 h**	0.568			0.494–0.642		0.071		0.609			0.542–0.676		0.002	
**PLR 0 h**	0.555			0.481–0.629		0.142		0.572			0.504–0.640		0.040	
**MLR 0 h**	0.521			0.447–0.595		0.598		0.584			0.516–0.652		0.017	
**NLR 48 h**	0.840			0.789–0.891		<0.0001		0.867			0.825–0.909		<0.0001	
**SII 48 h**	0.786			0.729–0.843		<0.0001		0.796			0.744–0.848		<0.0001	
**dNLR 48 h**	0.812			0.758–0.866		<0.0001		0.831			0.784–0.879		<0.0001	
**PLR 48 h**	0.730			0.667–0.794		<0.0001		0.740			0.682–0.798		<0.0001	
**MLR 48 h**	0.709			0.645–0.775		<0.0001		0.747			0.689–0.805		<0.0001	
**ΔNLR**	0.876			0.824–0.920		<0.0001		0.846			0.799–0.894		<0.0001	
**ΔSII**	0.834			0.781–0.887		<0.0001		0.793			0.739–0.847		<0.0001	
**ΔdNLR**	0.826			0.772–0.880		<0.0001		0.791			0.736–0.845		<0.0001	
**ΔPLR**	0.774			0.714–0.834		<0.0001		0.742			0.683–0.802		<0.0001	
**ΔMLR**	0.713			0.648–0.778		<0.0001		0.700			0.637–0.762		<0.0001	
**Need for IMV Prediction**														
		**AUC**	**95% CI**		***p* Value**		**Cut-off**		**Sn%**	**Sp%**		**PPV%**		**NPV%**
**ΔNLR**		0.876	0.824–0.920		<0.0001		>2		79.5	91.4		92.1		78
**ΔSII**		0.834	0.781–0.887		<0.0001		>340		79.5	80		83.3		75.7
**ΔdNLR**		0.826	0.772–0.880		<0.0001		>1		70.5	84.8		85.3		69.5
**ΔPLR**		0.774	0.714–0.834		<0.0001		>50		68.2	79		80.4		66.4
**ΔMLR**		0.713	0.648–0.778		<0.0001		>0.1		53.8	81.9		78.9		58.5
**Death Prediction**														
		**AUC**	**95% CI**		***p* Value**		**Cut-off**		**Sn%**	**Sp%**		**PPV%**		**NPV%**
**NLR 48 h**		0.867	0.825–0.909		<0.0001		>11		86.6	72.3		77.4		83.2
**SII 48 h**		0.796	0.744–0.848		<0.0001		>3700		71.8	70.8		72.9		69.7
**dNLR 48 h**		0.831	0.784–0.879		<0.0001		>6.93		80.3	70		74.5		76.5
**PLR 48 h**		0.740	0.682–0.798		<0.0001		>300		82.4	49.2		63.9		71.9
**MLR 48 h**		0.747	0.689–0.805		<0.0001		>0.64		60	80.8		77.3		64.8

Delta (Δ) values = 48 h value minus ICU admission value; statistical analysis was performed using the receiver operating characteristic (ROC) curves, AUC, and the statistical significance is highlighted for each parameter at 0 h, 48 h, and delta value depending on the studied event; NLR = neutrophil-to-lymphocyte ratio, dNLR = derived neutrophil-to-lymphocyte ratio, SII = systemic inflammation index, PLR = platelet-to-lymphocyte ratio, MLR = monocyte-to-lymphocyte ratio, 95% CI = 95% confidence interval, AUC = area under the curve, Sn = sensitivity, Sp = specificity, PPV = positive predictive value, NPV = negative predictive value, h = hours, IMV = invasive mechanical ventilation.

**Table 4:** Cox proportional hazards regression models for ΔNLR, ΔSII, ΔdNLR, ΔPLR, and ΔMLR for invasive mechanical ventilation need prediction.

**COX PH Regression: ΔNLR > 2, Univariate Analysis, *p* < 0.0001**			
**ΔNLR > 2**	***p* value**	**HR**	**95% CI for HR**
	<0.0001	6.88	4.47–10.60
**COX PH regression: ΔNLR > 2, Multivariate analysis, *p* < 0.0001, Method: Enter**			
**ΔNLR > 2**	***p* value**	**HR**	**95% CI for HR**
	<0.0001	5.05	3.06–8.33
**NIPPV**	0.002	1.88	1.27–2.77
**P/F ratio < 100 at 48 h**	0.028	1.52	1.05–2.21
**COX PH regression: ΔSII > 340, Univariate analysis, *p* < 0.0001**			
**ΔSII > 340**	***p* value**	**HR**	**95% CI for HR**
	<0.0001	5.05	3.30–7.74
**COX PH regression: ΔSII > 340, Multivariate analysis, *p* < 0.0001, Method: Enter**			
**ΔSII > 340**	***p* value**	**HR**	**95% CI for HR**
	<0.0001	3.56	2.21–5.74
**NIPPV**	<0.001	2.04	1.38–3.00
**P/F ratio < 100 at 48 h**	0.003	1.76	1.21–2.55
**C-Reactive Protein 48 h**	0.031	1.002	1.000–1.004
**COX PH regression: ΔdNLR > 1, Univariate analysis, *p* < 0.0001**			
**ΔdNLR > 1**	***p* value**	**HR**	**95% CI for HR**
	<0.0001	4.03	2.76–5.89
**COX PH regression: ΔdNLR > 1, Multivariate analysis, *p* < 0.0001, Method: Enter**			
**ΔdNLR > 1**	***p* value**	**HR**	**95% CI for HR**
	<0.0001	2.61	1.70–4.01
**NIPPV**	<0.001	2.01	1.36–2.95
**P/F ratio < 100 at 48 h**	<0.001	1.96	1.34–2.85
**COX PH regression: ΔPLR > 50, Univariate analysis, *p* < 0.0001**			
**ΔPLR > 50**	***p* value**	**HR**	**95% CI for HR**
	<0.0001	3.04	2.10–4.39
**COX PH regression: ΔPLR > 50, Multivariate analysis, *p* < 0.0001, Method: Enter**			
**ΔPLR > 50**	***p* value**	**HR**	**95% CI for HR**
	0.001	1.95	1.29–2.93
**NIPPV**	<0.001	2.03	1.38–2.98
**P/F ratio < 100 at 48 h**	<0.0001	2.15	1.48–3.13
**COX PH regression: ΔMLR > 0.1, Univariate analysis, *p* < 0.0001**			
**ΔMLR > 0.1**	***p* value**	**HR**	**95% CI for HR**
	<0.001	2.59	1.84–3.66
**COX PH regression: ΔMLR > 0.1, Multivariate analysis, *p* < 0.0001, Method: Enter**			
**ΔMLR > 0.1**	***p* value**	**HR**	**95% CI for HR**
	0.004	1.73	1.19–2.51
**NIPPV**	0.002	1.89	1.28–2.80
**P/F ratio < 100 at 48 h**	<0.0001	2.17	1.49–3.16
**C-Reactive Protein 48 h**	0.014	1.003	1.001–1.005

Only the variables identified as independent risk factors for invasive mechanical ventilation requirement were listed. For the studied hematological indices, results from univariate analysis are also listed. In each regression model the possible confounders were NIPPV, P/F ratio < 100 at 48 h, C-reactive protein at 48 h, Charlson Comorbidity Index, diabetes mellitus, and age > 60 years. All the variables were introduced in the regression analysis using the enter method. NLR = Neutrophil-to-lymphocyte ratio, dNLR = derived neutrophil-to-lymphocyte ratio, SII = systemic inflammation index, PLR = platelet-to-lymphocyte ratio, MLR = monocyte-to-lymphocyte ratio, NIPPV = non-invasive positive pressure ventilation, 95% CI = 95% confidence interval, PH = proportional hazards, HR = hazard ratio.

**Table 5:** Cox proportional hazards regression models for NLR, SII, dNLR, PLR, and MLR for death prediction.

**COX PH Regression: NLR > 11, Univariate Analysis, *p* < 0.0001**			
**NLR > 11**	***p* value**	**HR**	**95% CI for HR**
	<0.0001	4.6	2.80–7.56
**COX PH regression: NLR > 12, Multivariate analysis, *p* < 0.0001, Method: Enter**			
**NLR > 11**	***p* value**	**HR**	**95% CI for HR**
	0.003	2.25	1.31–3.86
**HAIs**	<0.001	2.31	1.58–3.40
**P/F ratio < 125 at 48 h**	<0.0001	1.97	1.34–2.87
**Higher respiratory support**	<0.0001	3.48	2.30–5.29
**COX PH regression: SII > 3700, Univariate analysis, *p* < 0.0001**			
**SII > 3700**	***p* value**	**HR**	**95% CI for HR**
	<0.001	2.44	1.68–3.54
**COX PH regression: SII > 3700, Multivariate analysis, *p* < 0.0001**			
**SII > 3700**	***p* value**	**HR**	**95% CI for HR**
	0.01	1.68	1.13–2.49
**HAIs**	<0.0001	2.3	1.56–3.39
**P/F ratio < 125 48 h**	<0.001	2.10	1.43–3.09
**Higher respiratory support**	<0.0001	3.76	2.50–5.62
**COX PH regression: dNLR > 6.93, Univariate analysis, *p* < 0.0001**			
**dNLR > 6.93**	***p* value**	**HR**	**95% CI for HR**
	<0.0001	3.44	2.26–5.24
**COX PH regression: dNLR > 6.93, Multivariate analysis, *p* < 0.0001, Method: Enter**			
**dNLR > 6.93**	***p* value**	**HR**	**95% CI for HR**
	0.005	1.89	1.2–2.98
**HAIs**	<0.0001	2.26	1.54–3.34
**P/F ratio < 125 at 48 h**	0.003	1.88	1.28–2.75
**Tocilizumab**	0.041	0.52	0.28–0.97
**Higher respiratory support**	<0.0001	3.73	2.48–5.62
**COX PH regression: PLR > 300, Univariate analysis, *p* < 0.0001**			
**ΔPLR > 300**	***p* value**	**HR**	**95% CI for HR**
	<0.0001	2.36	1.53–3.64
**COX PH regression: PLR > 300, Multivariate analysis, *p* < 0.0001, Method: Enter**			
**PLR > 300**	***p* value**	**HR**	**95% CI for HR**
	0.025	1.66	1.04–2.64
**HAIs**	<0.0001	2.22	1.50–3.28
**P/F ratio < 125 at 48 h**	<0.001	2.01	1.38–2.94
**Higher respiratory support**	<0.0001	3.50	2.35–5.26
**COX PH regression: MLR > 0.64, Univariate analysis, *p* < 0.0001**			
**MLR > 0.64**	***p* value**	**HR**	**95% CI for HR**
	<0.0001	2.38	1.70–3.33
**COX PH regression: MLR > 0.64, Multivariate analysis, *p* < 0.0001, Method: Enter**			
**MLR > 0.64**	***p* value**	**HR**	**95% CI for HR**
	0.048	1.49	1.003–2.20
**HAIs**	<0.001	2.19	1.48–3.22
**P/F ratio < 125 at 48 h**	0.001	1.93	1.32–2.81
**Higher respiratory support**	<0.0001	3.55	2.40–5.24

Only the variables identified as independent risk factors for invasive mechanical ventilation requirement were listed. For the studied hematological indices, results from univariate analysis are also listed. In each regression model the possible confounders were higher respiratory support needed, healthcare acquired infections, P/F ratio < 125 at 48 h, C-reactive protein at 48 h, Charlson Comorbidity Index, SOFA score at 48 h, tocilizumab therapy, diabetes mellitus, and age > 60 years. All the variables were introduced in the regression analysis using the enter method. NLR = Neutrophil-to-lymphocyte ratio, dNLR = derived neutrophil-to-lymphocyte ratio, SII = systemic inflammation index, PLR = platelet-to-lymphocyte ratio, MLR = monocyte-to-lymphocyte ratio, SOFA = Sequential Organ Failure Assessment, 95% CI = 95% confidence interval, PH = proportional hazards, HR = hazard ratio, HAIs = hospital acquired infections.

## Data Availability

The data presented in this study are available on request from the corresponding author.
